# Type I error control for cluster randomized trials under varying small sample structures

**DOI:** 10.1186/s12874-021-01236-7

**Published:** 2021-04-03

**Authors:** Joshua R. Nugent, Ken P. Kleinman

**Affiliations:** Department of Biostatistics and Epidemiology, School of Public Health and Health Sciences, University of Massachusetts Amherst, 715 North Pleasant Street, Amherst, 01003 Massachusetts USA

**Keywords:** Linear mixed models, Wald test, Likelihood ratio test, Type I error

## Abstract

**Background:**

Linear mixed models (LMM) are a common approach to analyzing data from cluster randomized trials (CRTs). Inference on parameters can be performed via Wald tests or likelihood ratio tests (LRT), but both approaches may give incorrect Type I error rates in common finite sample settings. The impact of different combinations of cluster size, number of clusters, intraclass correlation coefficient (ICC), and analysis approach on Type I error rates has not been well studied. Reviews of published CRTs find that small sample sizes are not uncommon, so the performance of different inferential approaches in these settings can guide data analysts to the best choices.

**Methods:**

Using a random-intercept LMM stucture, we use simulations to study Type I error rates with the LRT and Wald test with different degrees of freedom (DF) choices across different combinations of cluster size, number of clusters, and ICC.

**Results:**

Our simulations show that the LRT can be anti-conservative when the ICC is large and the number of clusters is small, with the effect most pronouced when the cluster size is relatively large. Wald tests with the between-within DF method or the Satterthwaite DF approximation maintain Type I error control at the stated level, though they are conservative when the number of clusters, the cluster size, and the ICC are small.

**Conclusions:**

Depending on the structure of the CRT, analysts should choose a hypothesis testing approach that will maintain the appropriate Type I error rate for their data. Wald tests with the Satterthwaite DF approximation work well in many circumstances, but in other cases the LRT may have Type I error rates closer to the nominal level.

## Background

In cluster-randomized trials (CRTs), also called group randomized trials, subjects are organized in groups. These groups, rather than the subjects directly, are randomized to the trial interventions [1]. In these studies, outcomes within a cluster – for example, patients within hospitals or students within classrooms – are almost certainly correlated with one another. This clustering complicates data analysis because the common regression assumption that observations are independent is violated. When the response variable of interest is continuous, linear mixed models (LMMs), which require that observations are independent only after conditioning on cluster membership, are a common approach to the data analysis. CRTs are a widely used experimental design (see for example [2–4]), and LMMs are an attractive option for data analysis. Some reasons for this attractiveness are that LMMs are robust to certain missing data mechanisms and can flexibly accommodate nested levels of clustering and/or varying cluster sizes [5].

Generalized linear mixed models (GLMMs) extend the approach to non-Gaussian data, such as binary, count, or multinomial outcomes. Issues we discuss in this paper may arise in these settings as well, though use of GLMMs introduces additional issues such as the choice of modeled distribution, link function, and the approximation of an intracluster correlation coefficient (ICC) with the natural parameters of that distribution. We do not investigate GLMMs in this article.

When fitting LMMs to CRT data, inference on parameters depends on asymptotic results, and in settings where the number of clusters is small they can generate Type I error (TIE) rates well above or below the nominal level [6]. All frequentist null hypothesis significance testing (NHST) theory depends on tests having the nominal size – a test with a nominal 5% error rate should produce false rejections 5% of the time. If not, data analysts in a CRT could be led to inappropriate conclusions when evaluating a treatment effect using NHST; for example, producing too many false positives or false negatives. Analysts evaluating associations using confidence intervals rather than null hypothesis significance testing may also be misled if asymptotic parameter distributions are incorrect with small samples.

Unfortunately, small cluster counts are not uncommon in the literature, because it is often more expensive to add more clusters to a study than more individuals to a cluster. Despite common heuristics such as ‘at least 30 units at each level of analysis’ [7], CRTs often have as few as 20 clusters. For example, a review of 100 CRTs [8] found 37% with fewer than 20 clusters and minimal reporting of any small-sample corrections employed.

Some limited investigations of the problems with (G)LMM small sample inference have been conducted. Pinheiro and Bates [6] examined a very restricted parameter space, while Schluchter and Elashoff [9] reviewed the issue from a slightly different angle, examining approaches for longitudinal data with different covariance structures, which have different interpretations than a typical CRT. Several studies [10–13] suggested improving small-sample inference by applying the Bartlett correction [14], also under a smaller set of parameters than we apply here. However, as far as we are aware there is no simple way for data analysts to implement the Bartlett correction in SAS or R.

Other studies [15–17] examine issues around small numbers of clusters, but include both random intercepts and slopes, which may not be a structure that all CRTs utilize. Closer to our setting in this article, Leyrat et al. [18] evaluated the power and TIE rates of different degrees of freedom (DF) choices for LMMs with Wald hypothesis tests for CRT designs under various design factors. They found both conservative and anti-conservative results, depending on the DF method chosen. Kahan et al. [8] reviewed small sample issues, but limited investigation to a small set of parameters and methods. Johnson et al. [19] examined LMM TIE rates, but only for Wald tests with two DF choices, and did not break down their results by design factors. In the GLMM context, for binary outcomes only, Li and Redden [20] examined TIE rates under different DF choices and found that the rates varied widely by method and design factors.

The work discussed above either does not break down the small-sample problems by design factor combinations (the effect of the ICC may vary depending on the number of clusters and cluster size, for example), does not compare results to the likelihood ratio test, and/or examines a limited set of data-generating parameters. Our work aims to add to this literature by examining in more detail the TIE control of several LMM inference approaches in a variety of plausible CRT scenarios. We examine both likelihood ratio test and Wald test results, including different DF choices for the latter. We also vary cluster size, number of clusters, and intracluster correlation coefficient, looking at how results vary under the different approaches. We hope to provide enough detail to alert data analysts to the situations that may lead to incorrect TIE rates with LMMs, and give guidance on which methods have the best error control given those factors.

## Methods

We performed a Monte Carlo simulation study to examine the TIE control of different LMM inference approaches under varying, plausible CRT circumstances. First, we describe the statistical model in question and the difficulties with small-sample inference, then we outline our specific study design. For all data analysis in this article, we used the SAS/STAT 15.1 (SAS Institute Inc., Cary, NC) and R 3.6.0 (R Foundation for Statistical Computing) software packages.

### Model

We consider here a version of the linear mixed-effects model of Laird and Ware [21]:

1 $$  Y_{ij} = \mathbf{X}_{ij}^{T}\boldsymbol{\beta} + \mathbf{Z}_{ij}^{T} \boldsymbol{b}_{i} + \mathbf{\epsilon}_{ij}  $$

where *Y*_*ij*_ is a continuous response variable for individual *j* in cluster *i*, $X_{ij}^{T}$ are that individual’s covariates for a vector of fixed effect regression parameters $\boldsymbol {\beta }, Z_{ij}^{T}$ are the cluster-level values for a vector of random effects ***b***_*i*_ for cluster *i*, and *ε*_*ij*_ is the residual error of the observation. In our case, matching common practice in CRTs, we restricted the random-effects structure to include only a random intercept term, so the term $Z_{ij}^{T} \boldsymbol {b}_{i}$ reduces to *b*_0*i*_. We let *ε*_*ij*_∼*N*(0,*σ*^2^) for all individuals, and cluster-level variance *b*_0*i*_ was distributed $N\left (0, \sigma _{b}^{2}\right)$, with *b*_0*i*_ independent of *ε*_0*i*_. We further assumed that cluster size is uniform for all clusters, and that there are two treatment arms with an equal number of clusters in each arm, modeled with an indicator variable *x*_*i*_∈{0,1} for control or treatment arm, with *β*_1_ being the treatment effect. Thus, for the remainder of the article, our model is:

2 $$  Y_{ij} = \beta_{0} + \beta_{1} x_{i} + b_{0i} + \epsilon_{ij}  $$

### Impact of clustering on inference

In a CRT, there are typically two assumed sources of variability in outcomes: between-cluster, denoted here as $\sigma ^{2}_{b}$, and within-cluster, denoted as *σ*^2^. The marginal variance of $y_{ij} = \sigma _{b}^{2} + \sigma ^{2}$. One way of quantifying the amount of clustering is via the intra-cluster correlation coefficient (ICC) *ρ*, defined as $\frac {\sigma ^{2}_{b}}{\sigma ^{2}_{b} + \sigma ^{2}}$, or the proportion of total variance due to the cluster-level variability. If one were to incorrectly analyze the data using a linear model rather than a linear mixed model, standard errors for the coefficient estimates would have to be adjusted, since observations are correlated in violation of the model assumptions. An approximation of this adjustment, the *design effect* [22], is a multiplier for the sampling variance of the treatment effect estimator. It is defined as [(*n*−1)*ρ*+1], where *n* is the number of subjects per cluster. For example, with 10 observations per cluster and an ICC of.01, the design effect is 1.09, meaning that the treatment effect coefficient standard errors would have to be multiplied by roughly $\sqrt {1.09} \approx 1.04$ to account for clustering. However, with 100 observations per cluster and the same ICC, the standard error multiplier increases to $\sqrt {2} \approx 1.41$, and for 1000 observations per cluster it increases to $\sqrt {11} \approx 3.31$, meaning that even a very small ICC can drastically change inferences when the cluster size is large. This approximation demonstrates the necessity of accounting for between-cluster variation in the data analysis, even if the ICC is expected to be small.

### Inference with LMM fixed effect estimators

Two ways of fitting a linear mixed model are by maximum likelihood (ML) and restricted maximum likelihood (REML), and most major statistical software packages can perform estimation by either method. Inference about $\hat {\beta }_{1}$ can be made using the likelihood ratio test (LRT) if fitting via ML, or by a Wald test if fitting via REML. A third test based on the maximum likelihood, the score test, is rarely used in this setting and is not discussed here. The LRT compares the log-likelihood of a model without *β*_1_ (*ℓ*_0_) to a model that includes it (*ℓ*_1_), and the test statistic *λ*=−2(*ℓ*_0_−*ℓ*_1_) has a $\chi ^{2}_{p}$ distribution, asymptotically, with degrees of freedom *p* the difference in parameter dimension between the two models. In our case, as in many CRTs, there is one treatment effect parameter, so *p*=1. In general, the LRT is recommended over the Wald test, as its asymptotic properties are superior [23]. Unfortunately, the *χ*^2^ distribution may be a poor approximation of the distribution of *λ* when the amount of information in a sample, for example, cluster count, is small.

Alternatively, a Wald test statistic under the null hypothesis *H*_0_:*β*_1_=0 can be generated by dividing the estimated treatment effect by its standard error: $t^{*} =\hat {\beta }_{1} / SE(\hat {\beta }_{1})$. This value can then be compared to a central *t* distribution. Unfortunately, for many designs, it is unclear what the appropriate degrees of freedom (DF) for that distribution should be [24]. Choices include:

- Residual: *N*−*p*, where *N* is the total number of observations and *p* is the number of fixed-effects coefficients to be estimated in the model. In the CRT design assumed here, *p*=2. Since the number of observations is usually much larger than the number of parameters in the model, this will generate similar results to the ‘*t* as *z*’ approach described below.
- Between-within: The residual DF are partitioned into between-subject and within-subject groups, equivalent in this case to a one-way ANOVA decomposition, meaning *DF*=*K*−2, where *K* is the number of clusters.
- Satterthwaite approximation: This method, generalizing the ideas of Satterthwaite [25], is quite complex, but it essentially uses the variance of the *β*_1_ estimate in its calculation of the DF. For more detail, see McCulloch et al. [26], Ch. 6.
- Kenward-Roger approximation: This method [27] inflates the fixed and random effects variance-covariance matrix, and calculates Satterthwaite DF based on these inflated values. Under our model with one treatment effect, it generates DF equivalent to the Satterthwaite approximation.
- Infinite (‘*t* as *z*’): The statistic is compared to a standard normal distribution, equivalent to a *t* distribution with infinite DF.

### Alternative inferential approaches

The Wald and likelihood ratio tests are not the only options for generating confidence intervals and performing inference in CRTs. Bayesian methods have been implemented with mixed models [28, 29], but we do not include Bayesian methods in this analysis. Alternatively, confidence intervals for LMM fixed effects can be generated by a parametric, semi-parametric, or non-parametric bootstrap. All are computationally intensive and require careful implementation due to the clustered nature of the original sample, so we chose not to investigate those approaches, though the parametric boostrap has been recommended by some authors [30].

### Data generation

We generated clustered, balanced data sets from the null model

3 $$  y_{ij} = b_{0i} + \epsilon_{ij}  $$

for clusters *i*=1,2,...,*K* and individuals *j*=1,2,...,*N* within each cluster. The random intercept *b*_0*i*_ for cluster *i* was distributed $\sim N(0, \sigma _{b}^{2})$, and the residual error term *ε*_*ij*_∼*N*(0,*σ*^2^). *b*_0*i*_ and *ε*_*ij*_ were generated as independent pseudorandom variates. We also generated values of *x*_*ij*_ such that for clusters *i*=1,...*K*/2,*x*_*ij*_=0, and for *i*=*K*/2+1,...*K*,*x*_*ij*_=1. This variable represents the treatment indicator, though it was not used in the data generation, as there is no treatment effect under the null hypothesis.

For each data set, we then fit the model shown in equation (2) using SAS PROC MIXED and the **lme4** and **lmerTest** packages in R. The coefficient of interest in these fitted models, $\hat {\beta }_{1}$, represents the estimated treatment effect.

We gathered p-values for the $\hat {\beta }_{1}$ coefficients using the LRT and the Wald test using the various DF options. We assessed the rejection rate under each test for the null hypothesis that *β*_1_=0 with *α*=.05. Since the data-generating mechanism had a true *β*_1_ value of zero, this estimates the TIE rate for the nominal *α*=.05 level.

We performed our analysis on 10,000 simulated data sets for all possible combinations of the following data-generating parameters:

- total number of clusters *K*∈{10,20,40,100}, divided evenly among the two treatment arms
- subjects per cluster *N*∈{3,10,20,50}
- $\sigma _{b}^{2} \in \{0.001, 0.01, 0.02, 0.05, 0.1, 0.2, 0.5\}$
- *σ*^2^=1

In preliminary simulations, we tested several different magnitudes for $\sigma _{b}^{2}$ and *σ*^2^ that produced the same ICC, and found that they generated the same Wald and LR test statistics. Based on this, we simplified number of parameter combinations to investigate by fixing *σ*^2^ at 1 and only varying $\sigma ^{2}_{b}$.

### Determining *p*-values

Both PROC MIXED and **lme4** report $\hat {\beta }_{1}$ estimates, their associated standard errors, and *t*^∗^ statistics. This allows for easy testing of the $\hat {\beta }_{1}$ coefficient via a Wald test, fitting with REML. The *t*^∗^ statistics generated were compared to *t* distributions with three choices of DF: between-within, Satterthwaite/Kenward-Roger, and residual, as described earlier. We then collected the p-values and calculated TIE rates under the three DF choices.

Both software packages also allow for model fitting using ML, allowing for model comparison and p-value determination for $\hat {\beta }_{1}$ via the LRT. First, a null model (4) was fit, with the only fixed effect being an intercept term:

4 $$  y_{ij} = \beta_{0} + b_{0i} + \epsilon_{ij}  $$

Second, a model with an added fixed effect for *x*_*ij*_, as in model (2). The doubled difference in maximized log-likelihood was compared to a $\chi ^{2}_{1}$ distribution since there was a one-parameter difference in model dimension. P-values from the $\chi ^{2}_{1}$ distribution were collected and TIE rates calculated.

## Results

Both software packages generated identical $\hat {\beta }_{1}$ estimates and standard errors when fitting with REML, and identical differences in likelihoods when fitting with ML. Reported results are from SAS. In addition, since the Kenward-Roger and Satterthwaite approximations were indistinguishable in this setting, they are both labeled as “approximate.”

**Fig. 1:**
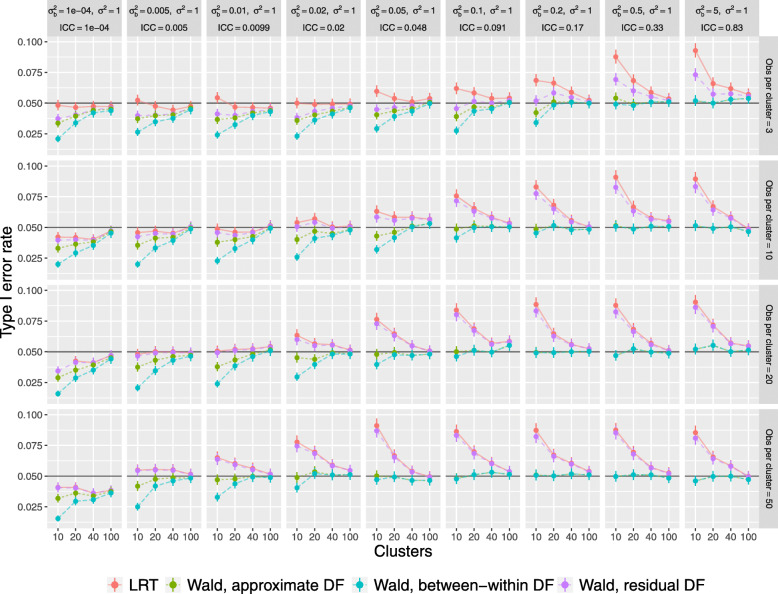
Relationship between Type I error rate and design factors

Results are displayed in Fig. 1. Under all approaches, departures from the nominal *α* level were most pronounced when the number of clusters is small.

When the number of observations per cluster is small, and there is a relatively small ICC, the LRT demonstrated appropriate TIE control. Regardless of the number of observations per cluster, the LRT is anti-conservative as the ICC rises. However, the anti-conservatism of the LRT was most apparent with smaller ICC when the number of observations per cluster was larger. Even with as many as 40 clusters and 50 observations per cluster, the LRT was noticeably anti-conservative once the ICC rose above.1. Worse, even when the ICC was very small (.01,.02), the LRT was anti-conservative with as few as 20 clusters of 50 observations per cluster.

As for the Wald tests, the between-within DF option led to conservative TIE rates when the ICC was small and/or the cluster size was small, but maintained the appropriate TIE rate with large clusters or a large ICC. The residual DF choice was less conservative in the case of a small ICC, but produced anti-conservative results as the ICC increased, and was more anti-conservative when the cluster size was large. Notably, depending on how the model is fit, the default method for determining DF in SAS may be ‘containment’, which under this study design leads to SAS assigning residual DF. Since this choice leads to the most anti-conservative results, it may be a concern for SAS analysts. The Satterthwaite approximation for our simulation estimated the DF as equal to the between-within DF in some cases and to residual DF in other cases, depending on the data set. This is why the TIE rates labeled “approximate” in Fig. 1 are bounded by those other two options.

We also tested the effect of an ICC of.09 generated with $\sigma ^{2}_{b} = 1$ and *σ*^2^=10 rather than the values discussed above. The results did not differ notably, which suggests that this pattern of TIE rate inflation with the LRT, as with the Wald test, is insensitive to the absolute size of the $\sigma ^{2}_{b}$ and *σ*^2^ values, only their relative size.

Finally, given the balanced nature of our data and the lack of other covariates, we could have used a *t*-test on the cluster means of each treatment arm to perform a hypothesis test. Using this approach, we achieved close to the nominal.05 alpha level in all cases. However, since most CRTs include covariates, a *t*-test would be inappropriate, and hence these results are omitted from the plot. The Wald test with the between-within DF choice is almost equivalent to this *t*-test [31], the only difference being that the LMM estimates two variances ($\hat {\sigma }_{b}^{2}$ and $\hat {\sigma }^{2}$), while the *t*-test only estimates their sum, leading to slighly different inferences.

## Conclusions

To our knowledge, the effect of different combinations of design factors and analysis approach on Type I error rates have not been examined comprehensively in previous reports. Our results show that none of the approaches meet the nominal alpha level in all cases examined, and the departures from the nominal level are directionally different based on the approach and data structure. Hence, there is no one-size-fits-all recommendation for data analysts in these small-sample cases.

The likelihood ratio test, based on an asymptotic *χ*^2^ distribution, does not perform well in these finite-sample cases, especially when the clusters contain many observations. This extends other studies that found the LRT to be anti-conservative [6, 32] in smaller explorations of the possible parameter combinations.

Alternatively, with a Wald test, some choices of DF, such as between-within or the data-adaptive Satterthwaite, can avoid anti-conservatism. However, a tradeoff exists, as they are too conservative when the ICC, the number of clusters, and/or cluster size is small.

After collecting our TIE rate results as outcomes, we formally tested the interactions between our design factors, using a three-way ANOVA within each analysis type and breaking the 10,000 simulations of each condition into 10 sets of 1,000, giving 10 outcomes per condition. Most of these three-way interactions were statistically significant, and given the strong patterns see in Fig. 1, we expect that we could show significance of all the interactions if we grew the number of simulations arbitrarily.

The results here suggest that data analysts should choose an approach that best suits their data. For example, if the ICC is expected to be small and the number of observations per cluster is small, the likelihood ratio test should perform well. For cases where the number of observations per cluster is large, a Wald test with the Satterthwaite DF approximation is better, though it can be conservative in some situations.

One perhaps unsatisfying conclusion is that analysts may want to generate their own small simulation studies to evaluate different approaches before fitting their final data models, since they will likely know the model structure, number of clusters, and cluster size by that point.

Finally, we caution analysts to be careful when using default setting in software. For example, with Wald tests, SAS PROC MIXED may default to the poorly-performing residual DF choice, and the **lmerTest** package in R defaults to the Satterthwaite approximation, which may be too conservative in some cases.

It is unclear how aware data analysts may be about the small-sample problems that may arise in making inference from mixed models. A review of linear mixed model applications in education and social sciences [33] found minimal reporting of estimation and inference methods and assumptions, and that cluster sizes could be as low as 2 and the number of clusters as low as 8. Our own review, and that of Kahan et al. [8], confirmed that small cluster counts are not unusual in biomedical settings as well. Therefore, we hope this will provide analysts with some recommendations of which approaches control TIE at appropriate rates under different circumstances, and we encourage more reporting of DF choices and analytic methods in CRT publications.

Our results, while limited to models with one random intercept, are in concordance with comparable LMM simulation studies with similar data-generating parameters but including random slopes [15–17], though only Luke [15] explored the same range of DF options considered here.

Given that small sample sizes are not uncommon in CRT literature, there is need for more investigation of which methods control TIE in other contexts. One limitation of our result is that we did not include any scenarios with repeated measures (for example, baseline, post-treatment, and follow-up), which are common in biomedical settings, and deserve similar scrutiny. Additionally, more parameters could have been added to the simulations, such as unbalanced cluster sizes or varying ICC by treatment arm. Previous simulation studies [34] demonstrated that unbalanced cluster sizes can result in inflated TIE rates. We suspect that the relatively good performance of the approximate DF will persist in these unbalanced cases.

Another potential avenue for exploration, following on the work of Li and Redden [20], would be to examine TIE rates under Wald tests and the LRT for GLMMs, in particular binomial, Poisson, and negative binomial-distributed outcomes, including various link functions. Further, a generalized ICC has been derived [35] and validated [36] for the negative binomial distribution, so the analysis could be replicated in a straightforward way. Type II errors may also be a concern for researchers, and investigating the role of different analytic methods on these could be an area for future work. Finally, the impact of these data/approach effects on statistical power should be determined so that analysts can make appropriate sample size calculations during the design phase of a CRT.

## Data Availability

The datasets generated and analysed during the current study are available in the following repository: https://github.com/joshua-nugent/joshua-nugent.github.io/tree/master/missingtie.

## Abbreviations

CRT: Cluster randomized trial
DF: Degrees of freedom
GLMM: Generalized linear mixed model
ICC: Intraclass correlation coefficient
LMM: Linear mixed model
LRT: Likelihood ratio test
ML: Maximum likelihood
REML: Restricted maximum likelihood
TIE: Type I error
